# Primary laryngeal manifestation in precursor T-cell acute lymphoblastic leukemia

**DOI:** 10.3892/ol.2014.2800

**Published:** 2014-12-12

**Authors:** LIN WANG, YAN MA, BOBIN CHEN, FENG TANG, XIAOPING XU, GUOWEI LIN

**Affiliations:** 1 Department of Hematology, Huashan Hospital, Fudan University, Shanghai 200040, P.R. China; 2 Department of Pathology, Huashan Hospital, Fudan University, Shanghai 200040, P.R. China

**Keywords:** laryngeal lymphoma, primary, leukemia, non-Hodgkin’s lymphoma

## Abstract

Primary involvement of the larynx in non-Hodgkin’s lymphoma (NHL) is rare. Early symptoms are non-specific and thus, it is difficult to diagnose. In the present study, the case of a 52 year-old male with hoarseness due to diffuse T-cell lymphoma as the first manifestation of precursor T-cell acute lymphoblastic leukemia is presented. Subsequent to treatment with three cycles of the etoposide and cytosine arabinoside (EA) and one cycle of the EA+L-asp chemotherapy regimens, the patient achieved complete remission. A series of consolidation therapy courses were performed subsequently. At present, the patient remains disease-free, indicating that the treatment was effective. Primary involvement of the larynx in NHL is rare. Symptoms in the early stage are subtle and non-specific and thus, diagnosis is difficult to establish. This type of tumor requires special diagnostic and therapeutic attention.

## Introduction

Non-Hodgkin lymphomas (NHLs) are a large group of cancers of lymphocytes. NHL can occur at any age and is often marked by lymph nodes that are larger than normal, fever and weight loss (1). Primary involvement of the larynx in NHL is rare, accounting for <1% of all laryngeal tumors, worldwide (2). To the best of our knowledge, <100 cases of primary laryngeal NHL have been reported, worldwide. In these cases, the most common site of involvement was the supraglottic region. Involvement of the glottis and subglottic region were rarely reported and cases with diffuse involvement of the whole larynx were extremely rare (2). The standard first-line chemotherapy regimens for the majority of cases of aggressive NHL include cyclophosphamide, doxorubicin, vincristine and prednisolone. Although the majority of NHL patients are responsive to the initial chemotherapy, 40–60% of patients do not achieve a complete response following first-line chemotherapy (3).

In the present study, the case of a 52 year-old male with hoarseness due to diffuse T-cell lymphoma as the first manifestation of precursor T-cell acute lymphoblastic leukemia (T-ALL) is presented. Written informed consent was obtained from the patient.

## Case report

**Figure 1:**
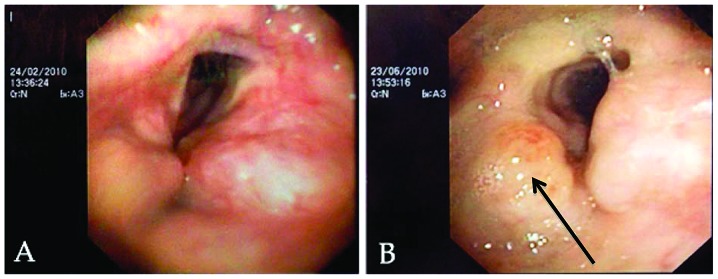
(A) Fiber laryngoscopic image revealing swollen laryngeal ventricles and false vocal cords. (B) Follow-up fiber laryngoscopic image revealing neoplasms in the ventricles of the larynx (arrow).

**Figure 2:**
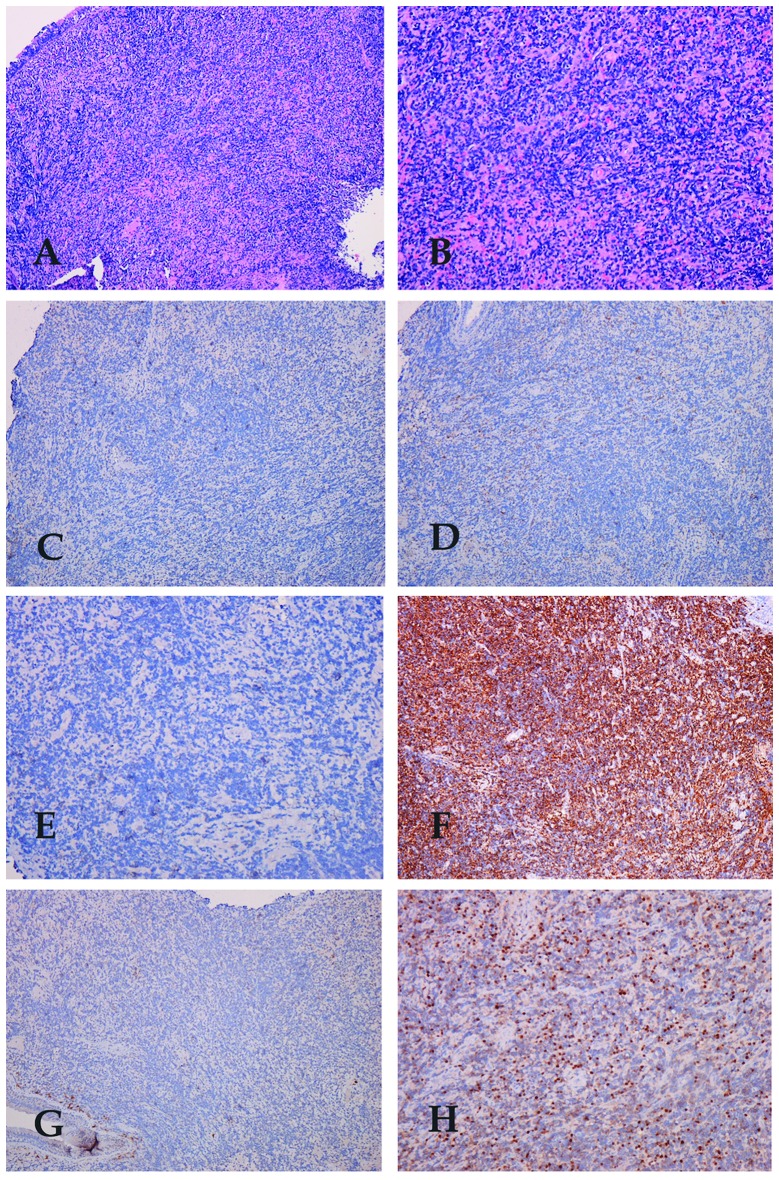
Immunohistochemical staining revealed (A) laryngeal mucosa with diffuse lymphocytic infiltration (stain, HE; magnification, ×40), and (B) lymphoepithelial lesions (stain, HE; magnification, ×100). Neoplastic lymphoid cells were negative for (C) CD5 (stain, HE; magnification, ×40), (D) CD10 (stain, HE; magnification, ×40) and (E) CD23 (stain, HE; magnification, ×100), positive for (F) CD43 (stain, HE; magnification, ×40), negative for (G) CD79 (stain, HE; magnification, ×40) and positive for (H) terminal deoxynucleotidyl transferase (stain, HE; magnification, ×100). HE, hematoxylin and eosin.

In August 2010, a 52-year-old male was admitted to Huashan Hospital, Fudan University (Shanghai, China) due to progressive hoarseness subsequent to overuse of the larynx ten months previously. Laryngoscopy had been performed at another hospital in December 2009, which revealed chronic swollen congestive vocal cords with a smooth surface. Computed tomography (CT) scan and blood tests revealed normal results. The patient was subsequently diagnosed with laryngitis. However, anti-inflammatory therapy did not improve the patient’s symptoms. Two months later, the patient underwent an additional laryngoscopy and biopsy. The laryngoscopy revealed swollen laryngeal ventricles and false vocal cords. The pathology of the laryngeal ventricles demonstrated chronic mucositis with diffuse infiltration of lymphoid tissue. The patient was diagnosed with chronic laryngitis, however, subsequent treatment with antibiotics did not alleviate the patient’s symptoms. In June 2010, routine blood tests revealed a red blood cell (RBC) count of 4.19×10^12^/l (normal range, 4.32–5.72×10^12^/l), a hemoglobin (Hb) level of 122 g/l (normal range, 135–175 g/l), a white blood cell (WBC) count of 11.6×10^9^/l (normal range, 4–10×10^9^/l) and a platelet (PLT) count of 119×10^9^/l (normal range, 100–300×10^9^/l). Additionally, the neutrophil granulocyte and lymphocyte percentages were identified to be abnormally low (20%) and abnormally high (78%), respectively, indicating hematologic abnormality. The hoarseness worsened and thus, endoscopy was performed, which revealed neoplasms in the two ventricles of the larynx (Fig. 1). Pathology of biopsy specimens obtained from the ventricular bands revealed neoplastic transformation of the lymphoid tissue. Immunohistochemistry revealed the cells to be positive for leukocyte common antigen, lysosome-associated glycoprotein cluster of differentiation (CD)68, CD3, terminal deoxynucleotidyl transferase and CD43 and negative for L26, CD79, CD56, granzyme B, perforin, latent membrane protein-1, Vimentin, CD5, CD10, CD23 and cytokeratin (Fig. 2). A CT scan of the neck revealed hyperplasia of the soft tissues in the supraglottic, glottis and subglottic regions. Three groups of lymph nodes in the submaxillary region were also involved.

In August 2010, the patient presented to the Department of Hematology, Huashan Hospital, Fudan University. Physical examination revealed lymphadenopathy and splenomegaly. Furthermore, blood tests revealed a RBC count of 3.55×10^12^/l, Hb level of 107 g/l, WBC count of 27.85×10^9^/l and PLT level of 110×10^9^/l, as well as an abnormally low percentage (13%) of neutrophil granulocytes and an abnormally high percentage (84%) of lymphocytes, indicating hematologic abnormality. A number of basket cells were also observed under the microscope (CX31-P; Olympus, Tokyo, Japan). In addition, a CT scan of the chest, abdomen and pelvis demonstrated superior mediastinum lymph node involvement. A bone marrow smear and biopsy indicated acute lymphoblastic leukemia, with 83.5% blasts. Immunophenotype analysis, which was determined by flow cytometry, revealed that precursor T cells accounted for 92% of the bone marrow total cell count and 85% of the peripheral blood cell count, indicating a definitive diagnosis of precursor T-cell ALL. The patient was administered with the VDCP chemotherapy regimen (2 mg vincristine, days 1,8,15 and 22; 60 mg daunorubicin, days 1–3 and 15–17; 1.0 g cyclophosphamide, days 1 and 15; 50 mg prednisolone, days 1–14). The patient experienced tachycardia after three days. Considering daunorubicin can interfere with the pumping action of the heart, epirubicin was administered instead of daunorubicin. Remission was not achieved after the first cycle of chemotherapy. The second chemotherapeutic regimen administered was EA (150 mg etoposide, days 1–4; 150 mg cytosine arabinoside, days 1–7). Three cycles of EA chemotherapy resulted in 17.5% blasts in the bone marrow. L-asparaginase was administered in the fourth EA regimen, which resulted in remission with blasts comprising 3% of the bone marrow. Laryngoscopy and CT scan were performed. No tumor tissue was identified in the larynx and the mediastinum involvement was also resolved. The patient then received six courses of consolidation therapy, including the EA + L-asparaginase (10,000 units L-asparginase, days 3 and 4) regimen and a high dose of methotrexate (3 g, day 1). At present, the patient remains disease free at the tumor site and lymph nodes.

## Discussion

Primary NHL of the larynx is rare. The age of onset for laryngeal lymphoma varies between four and 81 years, and previous studies have reported a male predominance (4). Patients with laryngeal NHLs usually present with progressive hoarseness, cough, stridor, dysphagia, a feeling of a foreign body and dyspnea. Early symptoms are nonspecific and thus, it is difficult to confirm the diagnosis. The main mass of the tumor is predominantly located in the supraglottic region, which contains lymphoid tissue in the lamina propria and ventricles. In certain cases, lymphoma may affect the glottis, however, the majority of cases occur in the subglottic or transglottic regions (2). The majority of tumors appear as a localized polyploidy sub-mucosal and nonulcerated swelling on endoscopy. CT scan or magnetic resonance imaging may aid assessment. Pathological and immunohistochemical analysis revealed plasmacytoma, mucosa-associated lymphoid tissue-lymphoma and diffuse large B-cell lymphoma to be the most common subtypes of NHL involving the larynx (5). T-cell laryngeal lymphoma is much less common than B-cell lymphoma (6).

However, at the time of diagnosis, the majority of patients already exhibit bone marrow or peripheral blood involvement, as well as mediastinal masses, lymphadenopathy and organomegaly. Uyttebroeck *et al* (7) reported that T-ALL and T-lymphoblastic lymphoma (T-LBL) represent precursor T-cell malignancies that reflect the various stages of T-cell development. A percentage of blasts >25% of total cells in the bone marrow differentiates acute lymphoblastic leukemia from lymphoblastic lymphoma (6). In the present case, precursor T cells accounted for 92% of the bone marrow of the patient and thus, T-ALL was diagnosed. T-ALL in adults is rare, accounting for ~25% of all adult ALL cases and it is more common in males than females. In the present study, the tumor cells were positive for CD3 and thus, according to the World Health Organization (WHO) classification of lymphoid neoplasms guidelines (8), the immunological subtype was diagnosed as T-IV (positive for surface CD3 and negative for CD1a, irrespective of other markers).

Treatments for T-ALL and T-LBL are different. It has been suggested that laryngeal T-LBL should be managed as a rare presentation of NHL. Radiotherapy and chemotherapy are the most common therapeutic strategies used for the treatment of primary laryngeal lymphomas. Surgery may be essential as the first line treatment if laryngeal obstruction or massive hemorrhage is identified (4). Kovac *et al* (6) reported a female patient with subglottic T-cell lymphoma as the only manifestation of precursor T-cell acute lymphoblastic leukemia. The patient underwent one cycle of chemotherapy with cyclophosphamide, doxorubicin, vincristine and prednisone, however, remission was not achieved. High doses of cytarabine (3 g) and mitoxantrone (15 mg) resulted in remission. The patient succumbed to an infection prior to auto transplantation surgery. In the present case, the patient did not respond to the first cycle of VDCP chemotherapy and thus, the regimen was changed. Following three cycles of the EA regimen and one cycle of the EA+L-asp regimen, precursor T cells accounted for 3% of the bone marrow of the patient. Laryngoscopy revealed complete remission of the tumor. Six consolidation therapy courses were performed subsequently. At present, the patient remains disease free, which indicates that the EA+L-asp regimen and treatment with a high dose of methotrexate are appropriate treatments for T-ALL.

To the best of our knowledge, this is the first report of a case of precursor T-cell lymphoblastic leukemia manifesting as diffuse involvement of the whole larynx. In summary, the symptoms during the early stage of the disease are subtle and non-specific and thus, diagnosis is difficult to establish. A number of deep biopsies, bone marrow biopsies and flow cytometry analysis are required to distinguish the tumor from inflammatory cells.

Primary involvement of the larynx in NHL is rare, accounting for <1% of all laryngeal tumors. The symptoms in the early stage are subtle and nonspecific and thus, diagnosis is difficult to establish. In the present study, a male patient with hoarseness due to diffuse T-cell lymphoma as the first manifestation of precursor T-ALL was reported. Following three cycles of the EA regimen and one cycle of the EA+L-asp regimen, the patient achieved complete remission. A series of consolidation therapy cycles were performed subsequently. At present, the patient remains disease-free, indicating that this treatment was effective.

In conclusion, clinical features are important in diagnosis. However, similar onset manifestations may be caused by different diseases. In this report, the onset clinical manifestation was hoarseness, which is often observed in patients with laryngeal tumors. In this case, based on the results of bone marrow biopsy and immunophenotype analysis, which revealed abnormal high percentages of precursor T cells, the diagnosis was precursor T-cell acute lymphoblastic leukemia, according to the 2008 WHO classification of lymphoid neoplasms (8).
